# Self-Reported Benefit and Satisfaction with a Beamforming Body-Worn Hearing Aid for Elderly Adults

**DOI:** 10.1155/2018/2046894

**Published:** 2018-11-01

**Authors:** Bradley McPherson

**Affiliations:** University of Hong Kong, Pok Fu Lam, Hong Kong

## Abstract

Hearing impairment is a leading cause of disability globally and is particularly prevalent in elderly populations. Hearing aids are commonly recommended to mitigate the adverse effects on communication associated with hearing loss. However, the acceptability of hearing aids to elderly individuals is low and the majority of potential users do not wear hearing aids. Most hearing aids are designed with a discreet form factor in mind, to minimize device visibility. Given the range of comorbidities associated with hearing loss in the elderly, this conventional form factor may not always be optimal. The present study examined the experiences of elderly individuals with a recently developed, unconventional, body-worn hearing instrument, the EasyHear™ Grand (Logital Co. Ltd., Hong Kong). The bilaterally fitted instrument incorporates large controls, a color display, beamforming sound processing, and Bluetooth capabilities. Forty-three elderly participants (mean age=71; range 46-88 years) were surveyed to gauge level of benefit and satisfaction with the device and opinions regarding the hearing aid. They were assessed using three standardized questionnaires (the International Outcome Inventory-Hearing Aids, the Profile of Hearing Aid Benefit, and the Client Oriented Scale of Improvement) and through open-ended, structured interviews. Participants rated their EasyHear device fitting highly for hours of use and improved quality of life and rated the device favorably for improved communication and benefit in background noise. A majority of users felt the device improved listening ability in their expressed area of greatest need, and also for their second highest prioritized area of greatest need. Less than 10% of users felt their listening was only occasionally or hardly ever improved when using the body-worn device. Benefit and satisfaction ratings with the EasyHear Grand were comparable to those in studies involving conventional form factor devices. Interviews highlighted areas where users felt the device could be improved—extra noise reduction, changes to device dimensions, receiver/eartip fit, and cableless technology were among the areas mentioned. Many participants valued smartphone linkage and Bluetooth capability. The EasyHear Grand, with its body-worn design and large, simple controls, was well accepted by the majority of participants. Hearing aids that break from conventional design formats may benefit many elderly individuals with hearing impairment and promote increased user acceptability.

## 1. Introduction

Hearing loss is one of the principal global causes of disability [1] and the prevalence of hearing loss is particularly high in the elderly. Hearing loss is the leading cause of disability among men 60 years and older and is the second most common cause of years lived with disability for women in this age group [2]. Almost one-third of all adults over the age of 65 years have some degree of hearing loss, with 226 million over 65 years having disabling hearing loss [3]. The most common cause of this hearing impairment is presbycusis—sensorineural hearing loss resulting from age-related pathologic events in the auditory system [4]. Presbycusis can have major adverse effects on quality of life due to reduced communicative abilities [5].

As yet, there is no pharmacological intervention for presbycusis and the most commonly advised treatment is use of an amplification device. This typically involves unilateral or bilateral hearing aid fitting. A wide range of amplification devices are available for individuals with presbycusis and over 15 million hearing aids are globally fitted each year [6], primarily to users with presbycusis. However, despite their widespread availability—at least in developed economies—the overall acceptability of hearing-aid use is not high [7–11]. The majority of elderly individuals with presbycusis do not wear hearing aids [12, 13] and it takes those who do wear hearing aids an estimated ten years to acquire amplification devices after their first awareness of hearing loss [14].

Present-day hearing aids have a limited range of form factors. The vast majority of hearing aids are based on behind-the-ear (BTE) or in-the-ear (ITE) designs. These designs may not be appropriate for all elderly individuals with hearing loss. Many elderly persons with hearing impairment have comorbidities such as impaired vision, limited sensation of touch, limited range of movement, and dementia [10, 15–18]. Such conditions may make aspects of a hearing aid that are commonly viewed as positives much less advantageous. For example, prioritizing “invisibility” in a device [19] may lead to a small BTE or ITE instrument that presents increased management issues for the user. Many prospective elderly hearing aid users have difficulties in performing basic tasks associated with device management, such as correctly inserting a hearing aid or manipulating a volume control [20–22]. In a survey of the reasons for nonadoption of a hearing aid among elderly persons with hearing loss a majority of respondents considered not understanding how to manipulate a hearing aid, volume control difficulties, and the small size of hearing aids to be barriers to purchase [23]. Negative perceptions of hearing aids can reduce potential usage [24].

For potential elderly amplification device users with such issues a larger instrument that is easier to see and manipulate has benefits [25]. The form factor usually taken by larger devices is a body-worn design. However, body-worn hearing aids comprise less than 1% of hearing aid market and are rarely offered to clients in developed economies [26]. There have been few changes over the past decades in the design of those body worn devices that are available. Body worn hearing aids are typically single channel monaural devices with conventional omnidirectional microphones and a limited range of sound processing options.

**Figure 1:**
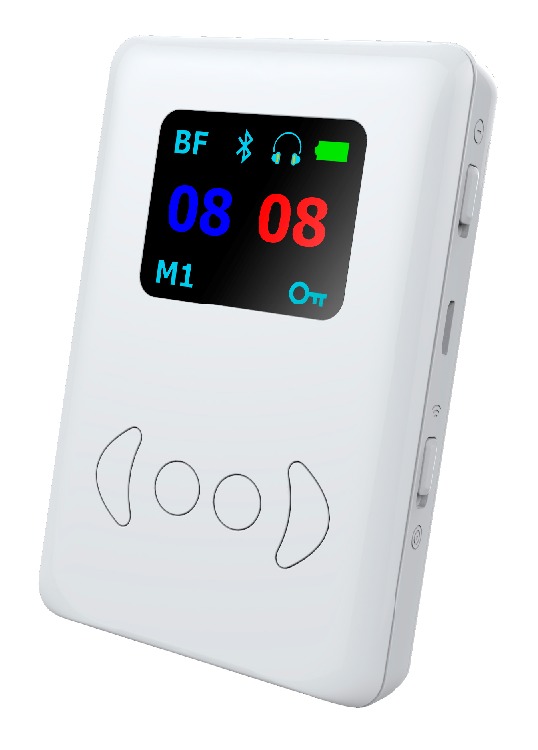
Front face of EasyHear Grand hearing aid.

A recently developed hearing aid, the EasyHear™ Grand (Logital Co. Ltd., Hong Kong), focuses on assisting persons with hearing impairment to combat real-world noise and enhance mid-range, face-to-face communication. The hearing aid was designed for a variety of users, but particularly for elderly clients who seek an easy to use and robust hearing device. The EasyHear Grand is a body-worn hearing instrument (92mm x 63.4mm x 16.3mm; 105.5 g) providing unilateral or dual, independent, right/left channel bilateral amplification. The hearing aid uses a 3.8V Li-ion rechargeable battery with a typical battery life of 10 hours and a 6-6.5 hour charging time. The EasyHear Grand has a frequency range of 300–8000 Hz with a maximum power output of 136 dB SPL at 900 Hz. The EasyHear device allows wearers to use a proprietary beamforming technology [27] that focuses on sound sources ± 30° in front of the listener, reduces noise from outside the frontal field, and preserves interaural cues. The hearing aid has adaptive noise cancellation ability, three automatic gain control kneepoints are available, and eight equalization bands are provided. The EasyHear Grand (Figure 1) is an unconventional hearing aid—it has a 4.57 cm color OLED screen to display device settings and comes with Bluetooth, smartphone linkage, and FM radio functions. The device may be appropriate for many older clients because of the large size of the controls and OLED display, simplicity of the controls, large Li-ion rechargeable battery, and smartphone compatibility.

Anecdotal reports from EasyHear clients suggested that the device improved listening ability for many of its users. However, no formal clinical studies had been conducted to evaluate the device with a senior client group. Indeed, in general, the effectiveness of nonconventional hearing aids is rarely evaluated [28].

One possible way to improve hearing aid acceptance is to collect information from the user perspective in the fitting trial period, particularly after user experience in everyday listening environments, and use this information to reflect upon future device adaptations and enhancements. This strategy has been considered essential since the early days of hearing aid provision [29, 30]. Self-reported outcome measurement tools for measuring benefit and satisfaction with hearing aids provide real-world information of hearing aid usage in a systematic way [31].

User benefit and satisfaction with the EasyHear Grand were investigated in the current study. Self-reported outcome measures—questionnaires and open-ended interviews—were completed before and after fitting to evaluate how effective the device may be with elderly users in everyday listening situations. Standardized questionnaires and open-ended interviews were employed to provide (a) ratings of benefit and satisfaction with the EasyHear Grand device and (b) an understanding of the elderly client experience with this type of hearing aid. The study aimed to consider this device in relation to the globally increasing need for effective solutions to presbycusis-related hearing loss.

## 2. Materials and Methods

The present study examined user benefit and satisfaction with the EasyHear Grand for individuals who had a bilateral hearing impairment, with moderate to severe mixed or sensorineural hearing loss in the better ear, and with or without previous experience with hearing amplification devices. The prospective, repeated-measures study was carried out over eight weeks with each participant. A set of standard, self-reported outcome measure questionnaires were used as well as an open-ended interview session to collect information regarding user benefit and satisfaction ratings with the EasyHear Grand. An eight-week final review point was chosen because outcomes from hearing aid fitting are stable after approximately six weeks post-fit [28].

### 2.1. Participants

A new EasyHear hearing device was provided to Cantonese-speaking persons with moderate to severe bilateral sensorineural hearing loss. Participants were recruited through EasyHear clinics at two localities in Hong Kong, China. Participants who agreed to join the program and who are within the inclusion criteria underwent diagnostic audiological assessment and cognitive screening assessment. They were prescribed with the device by a clinical audiologist based at the hearing clinics. Study participants were recruited (a) during a routine EasyHear Grand hearing aid first fitting session, or (b) by offer of a free trial with the EasyHear Grand. Participants were verbally informed about the aims of the study and written information was also provided. All participants visited their hearing clinic three times during the study, including a long-term follow-up visit eight weeks after initial EasyHear fitting. Assessment duration was approximately 60 minutes per visit. Written consent was obtained from each participant and the research protocol was approved by the Human Research Ethics Committee, The University of Hong Kong, prior to study commencement. A HKD 200 payment was made to subjects who completed the three visits, as compensation for travel costs.

Formal inclusion criteria were (1) above 40 years of age at commencement of the study; (2) Cantonese as the primary or secondary spoken language; (3) mixed or sensorineural hearing loss with the pure tone average at 0.5, 1, and 2 kHz in the better hearing ear > 40 dBHL and ≤ 90 dBHL; (4) both first time users and experienced hearing aid users; (5) adequate physical and cognitive status to enable the individual to follow the study protocol; and (6) willing to participate and to comply with all requirements of the protocol. Formal exclusion criteria were (1) cognitively impaired; (2) unwilling or unable to complete all testing sessions; (3) asymmetrical hearing loss with a difference of pure tone average between the left and the right ear of more than 30 dBHL; (4) purely conductive hearing loss in one or both ears; and (5) previous user of an EasyHear device.

### 2.2. Procedures

Initial collection of participant demographic details, and medical and hearing health history, was followed by recording of pure tone audiometry results from assessment at the clinic. Then baseline data were compiled using the following assessments: Montreal Cognitive Assessment (MoCA) test (Hong Kong version); International Outcome Inventory for Hearing Aids (IOI-HA) questionnaire; Chinese Profile of Hearing Aid Benefit (C-PHAB) questionnaire; and the Client Oriented Scale of Improvement COSI questionnaire. Visit 1 was split into 2 parts, with the first part (Visit 1a) focused on collection of participant demographic details, medical and hearing health history, audiometric and cognitive (MoCA) assessment, and hearing aid fitting, and the second part (Visit 1b), two to seven days later, focused on completing the COSI, IOI-HA, and C-PHAP questionnaires. The IOI-HA, C-PHAB, and COSI questionnaires were administered again at the second and final visits. At the final visit participants were also asked a series of open-ended interview questions about their experience with the EasyHear Grand. All responses were encouraged, whether they were positive or negative about the device.  Table 1 outlines the assessment sequence over the visits to the clinic.

The Montreal Cognitive Assessment (MoCA) test is a 30-item questionnaire used to assess mild cognitive impairment [32, 33]. The test has been adapted for use in over 40 languages including Hong Kong Chinese [34] and assesses six cognitive domains (short term memory recall, visuospatial abilities, executive function, attention, language, and orientation to time and space). Cognitive impairment was defined as a MoCA score of ≤25 points out of total score, a standard pass/fail criterion [34]. The International Outcome Inventory for Hearing Aids (IOI-HA) has been translated to over 20 languages [35] including Chinese and is a brief seven-item questionnaire that measures perceived use and benefit from a hearing aid, along with degree of remaining difficulties and lifestyle limitations, satisfaction with the device, impact of hearing loss on others, and changes in overall quality of life. The Cantonese version of the Profile of Hearing Aid Benefit (C-PHAB) questionnaire was also developed to quantify hearing aid performance [36]. The C-PHAB complements the IOI-HA by exploring outcomes in more detail, with a 42-item questionnaire that considers many situations where amplification may benefit the listener, including difficult listening environments and with unfamiliar talkers. The Client Oriented Scale of Improvement (COSI) questionnaire was the final measure of hearing aid fitting outcome. This is a less structured questionnaire—participants verbally identify listening situations that are personally important to them and rate how well they can hear in those situations with and without the hearing aid [37].

### 2.3. Device and Device Fitting

**Figure 2:**
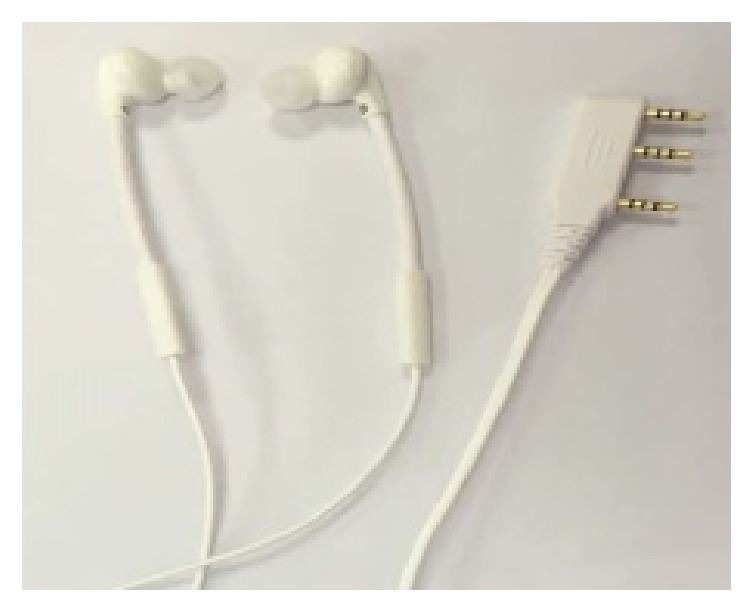
Bilateral earphone/microphone/input cable configuration for EasyHear Grand hearing aid. Note microphone position below each earphone.

**Figure 3:**
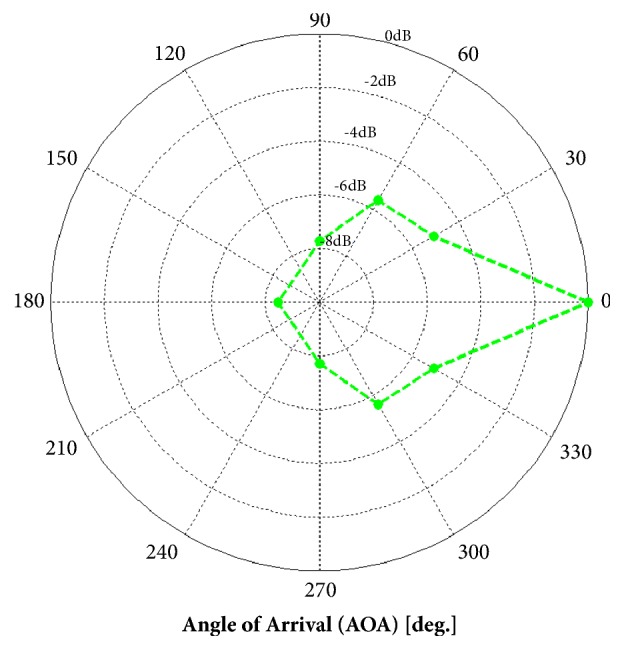
EasyHear Grand beamforming performance with device suspended in an undisturbed field (manufacturer supplied).

The EasyHear Grand is an air conduction amplification device intended to be fitted in a hearing health care setting and not purchased as an over-the-counter device. The fittings were all bilateral, with maximum output and frequency response characteristics prescribed for each individual ear using the manufacturer's proprietary fitting algorithm. Participants were mostly fitted with standard, closed-dome earmolds. If a closed-dome earmold did not fit well, or if acoustic feedback occurred, custom earmolds were manufactured for the participants. The elderly participants were allowed to freely control the device, including volume and listening mode selection (noise-cancelling plus beamforming or noise-cancelling only modes) during the fitting period. The hearing aid is typically placed in a shirt pocket or hung from a lanyard in front of the chest. A single, omnidirectional microphone is located on both the left and right earphone cables of the EasyHear Grand (Figure 2). The earphones house the system's miniature speakers. Shielded cable is used to connect the earphones/earmolds to the body of the device. The microphone signals provide input for proprietary beamforming algorithm processing, which takes place within the body of the device. The processed sound is a true stereo signal with separate left and right ear channels and retains interaural differences that enable localization cues. The extent of beamforming is indicated by the polar diagram shown in Figure 3, with approximately 5-9 dB maximum attenuation, depending on angle of arrival, reported by the manufacturer.

The test device was fitted at Visit 1 according to each participant's most recent pure tone audiometry results. The audiogram was entered into a proprietary desktop computer program. The software generated the initial fitting parameters (including volume, frequency response, and wide range dynamic compression). These custom settings were then entered into the EasyHear Grand. Fitting parameters were ear specific for this device as bilateral fittings leading to aided binaural hearing were standard. The hearing aids were fine-tuned at later visits if a feedback problem or discomfort from the amplified sound was noted. The test devices were fitted with one predefined listening program for the test period. To reduce the potential for bias effects, data at Visits 2 and 3 were obtained by university research assistants who were not affiliated with the EasyHear clinics and were known by the participants to be unaffiliated to the clinic.

### 2.4. Statistical Analyses

Both qualitative and quantitative analyses were undertaken. Quantitative statistical analyses for questionnaire test scores at Visit 1, Visit 2, and Visit 3 were carried out, along with qualitative thematic analysis of the open-ended interview responses obtained at Visit 3. A significance level of p<.05 was set for all quantitative analyses.

## 3. Results

### 3.1. Participant Information

Table 2 summarizes the characteristics of the 43 participants who completed the study over the data collection period, from December 2016 to December 2017. The participants joined the study after initial purchase of an EasyHear Grand (n=24) or after they were offered a free trial with the hearing aid (n=19). All participants had a bilateral sensorineural or mixed hearing loss and were all provided with devices using a bilateral fitting mode for binaural hearing function.

All participants passed the MoCA evaluation (i.e., had a score ≥ 26 out of 30 points). Nine potential participants were excluded from the study because they did not achieve a MoCA score of ≥ 26 points. Eight additional potential participants dropped out of the study before completion because they had left Hong Kong (n=1), could not comply with the revisit schedule (n=5), or returned the device because they feared losing it before the end of the study (n=2).

### 3.2. International Outcome Inventory: Hearing Aids

For each of the seven IOI-HA questions a score of five indicates the best possible outcome and a score of one indicates the worst. Domains are (1) use of the hearing aid, (2) benefit from the device, (3) residual difficulties when wearing the hearing aid, (4) overall satisfaction with the device, (5) residual participation restriction (how much does hearing loss still affect daily life), (6) impact on others, and (7) quality of life change. The mean overall IOI-HA scores were 3.59, 3.83, and 3.86 for Visits 1, 2, and 3, respectively. Mean scores for each of the seven question items and the overall score are shown in Table 3. A repeated measures ANOVA for total score (over 3 visits) was conducted. Results found a significant difference in IOI-HA total score over time, F(2,80)=10.53, p<0.001.

### 3.3. Cantonese Profile of Hearing Aid Benefit

Repeated measures ANOVAs were carried out for all four C-PHAB domains over the three visits with the participants wearing their EasyHear devices. Significant differences were found for ease of communication benefit (F(2,24)=5.2, p=.01), background noise benefit (F(2,42)=3.49, p=.04), and aversiveness benefit (F(1.44,33.03)=5.17, p=.02) across visits. The Reverberation questions were completely answered by only nine of the participants (see Table 4). Many of the participants were unfamiliar with some of the environments illustrated in these questions (such as large, reverberant spaces), as they were not commonly visited by this elderly group. Therefore, the Reverberation and Global Score (which includes the reverberation items) categories had low participant numbers and statistical analysis was not conducted.

### 3.4. Client Oriented Scale of Improvement

When asked to identify the main areas where they needed assistance from a hearing aid all participants (n=43) nominated at least one specific need, and virtually all could nominate a second need (42 participants). Over half the participants could nominate a third specific need (n=25) but only two participants nominated four specific areas they needed help with.

For specific need 1 the most common priorities were face-to-face communication, listening at a distance from a speaker, and telephone conversations. For specific needs 2 and 3, listening to speech in noise and watching television were often additional priorities. The range of needs expressed by participants at their first visit is summarized in Table 5.

The COSI questionnaire also allows participants to rate the final degree of improvement they feel their hearing aid has provided—from “hardly ever” any improvement (10% improvement only) to “almost always” improvement (95% improvement). The COSI results for the degree of improvement for main expressed needs and for specific needs 1, 2, and 3 at Visits 2 and 3 are summarized in Table 6. The majority of participants considered the EasyHear Grand to have improved their listening ability in their main situations of need “most of the time” or “almost always.”

### 3.5. Open-Ended Interview

At the final visit each participant was asked a series of interview questions about their experience with the EasyHear Grand. A summary of responses to the six questions asked is shown in Table 7. Responses were generally positive, with 41 out of 43 EasyHear users stating that their EasyHear device “improved audibility.” Twenty out of the 24 participants with previous hearing aid experience stated that the EasyHear device “sounded clearer” than their former hearing aid, 13 considered the EasyHear Grand to have “better noise reduction,” and 9 felt the Easy Hear Grand “sounded more natural.” A summary of the main participant responses to the interview questions is shown in Table 7. Twenty-seven out of 43 respondents liked the EasyHear Grand's ability to make “speech clearer.” Telephone linkage and Bluetooth functions were other features that were positively rated by numerous participants.

Participants commented on both the hardware and software features of the device and the most common suggestions for possible improvements are noted in Table 7. Related to the body-worn form of the device, 28 participants suggested that the device could be improved by “removing the wire,” indicating that for many clients the cable between device case and receiver/eartip inherent with a body-worn design was considered a negative factor. This was a more commonly mentioned negative point than the hearing aid's primary component, the device case itself. Only seven out of 43 participants felt that the device was “too big,” and no respondents expressed a preference for a device with a BTE/ITE form factor. Seven participants felt the receiver/eartip was “easy to fall” out of the ear—probably related to the device's usage of noncustom eartips for the majority of fittings. Other comments made by smaller numbers of participants were “device too expensive” (n=4); “faster battery charging” (n=3); “add a clip to the back” (n=2); “receiver should be over the head” (n=3); and “sound quality not good enough” (n=3).

## 4. Discussion

### 4.1. International Outcome Inventory: Hearing Aids

IOI-HA scores were evaluated to gauge the EasyHear Grand wearer experience. On average, participants used their hearing aid between 4 and 8 hours each day. Participants still had difficulty in the communication situation they most wanted improved hearing, but this difficulty was now only slight on average and participants generally now felt their hearing loss only slightly affected their everyday activities and that their family and friends were now only slightly bothered by their hearing loss. EasyHear Grand users felt that, overall, their device had made “quite a lot” of improvement to their enjoyment of life.

As the IOI-HA, PHAB, and COSI have been previously used, it was possible to compare EasyHear Grand group results with other studies. In the only directly comparable Hong Kong study [31] the mean group IOI-HA score at final interview was 3.82 out of a possible score of five. This was for 19 elderly persons (mean age = 73 years) with mild hearing loss who were fitted with a ReSound Avance mini BTE hearing aid that was preprogrammed with a frequency response for mild to moderate high frequency hearing loss (typical in elderly clients with presbycusis). This score indicates that for the overall IOI-HA measure the EasyHear Grand performed at least as well as the hearing aid used in the previous study. By the final visit, for frequency of use, 18 participants indicated they wore the device from 1 to 4 hours per day, 15 participants wore the device from 4 to 8 hours, and another 8 participants wore the hearing aid for over 8 hours per day. All 43 participants stated that the hearing aid was helpful in different situations and 31 reported little or no residual limitation when wearing the EasyHear Grand. All participants judged the device as “worth the trouble” of wearing. Similarly, comparison to a USA study of satisfaction and benefit in 154 experienced hearing aid wearers with a mean age of 77 years [38] shows consistently higher IOI-HA ratings for the present device, for most questionnaire items, than for the moderate to severely impaired US group. In comparison to an Australian survey of 1653 adults fitted with hearing aids in private practice clinics [39] the EasyHear overall mean score of 3.90 is almost identical to the Australian result of 3.91. These results (detailed in Table 8) suggest that satisfaction and benefit outcomes obtained from the EasyHear device were comparable to those obtained in other professional hearing aid fitting programs, across a range of studies involving hearing aid users with differing degrees of hearing loss.

Participants in less recent IOI-HA studies wore analog or early digital technology devices. It has been suggested that scores using more advanced devices may be higher [40]. Therefore, the EasyHear Grand results were also compared to a recent research report, based on over 100,000 hearing aid fittings carried out in Sweden in the period 2012-2016 [41]. These fittings were all carried out in professional dispensing clinics with a wide range of modern, conventional, digital hearing aids. The mean outcome score was 3.82—similar to the EasyHear Grand overall mean score of 3.86. Therefore, the EasyHear Grand's satisfaction and benefit scores are comparable to those noted in this and other clinical studies.

Mean IOI-HA scores showed slight but statistically significant improvement over time. Generally, only small changes in IOI-HA scores occurred between visits although a trend can be seen (in six out of seven questions) for scores to improve between initial fitting and final visit. This suggests that the EasyHear Grand users noted positive changes soon after they first experienced their changed auditory environment and they did not alter their opinions very much over the following months of hearing aid use.

### 4.2. Cantonese Profile of Hearing Aid Benefit

Three C-PHAB question areas were fully answered over all visits and significant improvements were noted in participant responses over the three visits for these areas—ease of communication benefit, background noise benefit, and aversiveness benefit. The reverberation area had low response numbers and therefore this area and the global score were not included in the final analysis. A previous Hong Kong study [31] found C-PHAB group scores for ease of communication benefit 0.10, background noise benefit 0.24, and aversiveness benefit 0.19 at the final interview. This compares to ease of communication benefit 0.47, background noise benefit 0.38, and aversiveness benefit 0.04, at the same interview in the EasyHear study. Note that the participants in the earlier study had mild hearing loss and it would be expected that they would gain less benefit from an amplification device compared to hearing aid users who had a greater degree of hearing loss. However, the aversiveness score was lower (poorer) for the EasyHear group. Possibly this is due to the group being fitted with a device with greater maximum power (the EasyHear Grand), and therefore the participants experiencing more high sound level inputs than they had been accustomed to, compared to the earlier study group wearing the lower gain ReSound Avance device.

### 4.3. Client Oriented Scale of Improvement

The specific needs mentioned by EasyHear wearers in the COSI questionnaire were typical of those expressed by most hearing clinic clients. The common priorities were face-to-face communications, listening at a distance from a speaker and telephone conversations, along with listening to speech in noise and watching television. For a somewhat younger group of clients, with milder hearing loss, the previous Hong Kong study [31] found communication (e.g., with family or others, or using telephone), entertainment (such as watching television), and a sense of security (e.g., listening to environmental sounds) were priorities. In that 2005 study the mean score for aided hearing improvement at final interview was 3.45 and for final hearing ability was 3.77 for first ranked specific need. Slightly higher scores were found in the EasyHear Grand group—3.8 for aided hearing improvement and 4.1 for final hearing ability at the final interview. Both studies are similar in that clients had access to a single type of hearing aid, either the EasyHear Grand or ReSound Avance. Similar results to the present study were found in a joint Argentina-Canada study of adults with a mean age of 68 years [42], with improvement and final scores both approximately 4. A Taiwanese report also notes similar COSI outcome scores for 59 hearing aid users between 65 and 80 years of age [43]. Both studies involved professional hearing aid fittings by audiologists with access to a wide range of conventional hearing aids. The EasyHear Grand results are, however, not as positive as those in a Welsh hospital-based study [44], which found a mean aided hearing improvement COSI score of 4.29 and mean final hearing ability score of 4.19. An Australian hearing clinic study [37] also recorded higher COSI scores, with a mean aided hearing improvement COSI score of 4.47 and mean final hearing ability score of 4.45. Both the Australian and Welsh groups were mainly elderly clients but both groups were fitted by professionals who had access to a wide range of hearing aids and fitting technology. The EasyHear device, while suitable for many of the elderly clients seen in this study due to its flexible fitting range, may not be able to satisfy the needs of all participants. It is to be expected that some participants have specific needs that may be better suited to other devices with different features or acoustic capabilities.

### 4.4. Open-Ended Interview

A substantial majority of EasyHear study participants felt the device improved their general hearing ability and made speech clearer—95% of users felt this was the case. Many participants also considered that the EasyHear Grand improved their ability to listen in noise (33%). Noise reduction was highlighted by participants as being a specific benefit of the hearing aid. Very few (only 5%) considered that the device made no difference to audibility or made listening a worse experience. Future research work to quantify expected improvements in speech perception using the EasyHear Grand beamforming technology would be useful. Speech perception studies with the EasyHear Grand in controlled quiet and noisy environments would provide a better understanding of the effects of the amplification system on elderly listeners with hearing loss.

Around a quarter of participants (n=11) mentioned Bluetooth functionality as a specific positive feature of the EasyHear Grand. This suggests that many of the elderly participants in this study were making use of this function to link with their smartphone or other devices. However, a number of participants also mentioned difficulty using the Bluetooth function and this suggests there is room for further device improvement or client education in this area.

Noise is a perennial problem for hearing aid users. The EasyHear Grand uses beamforming and other technology to reduce the impact of background noise on speech but—like all hearing devices—cannot eliminate noise entirely. Although many clients praised the EasyHear Grand's success in reducing background noise problems, noise was still a problem for listeners. The most frequent suggestion for improvement was for further “noise reduction.” Additionally, some participants felt that device size could be reduced and that there were problems with earphone stability and/or comfort in the ear. Further thought to earphone and earmold design may be useful. The increasing use of wireless technology in many electronic devices may have influenced the large number of respondents (n=28) who advised removing wires from future EasyHear models. Obviously a wireless instrument increases convenience for wearers but potential disadvantages may be involved, such as increased power consumption and device size. Several participants suggested adding a clip on the rear face of the EasyHear Grand for easier attachment to clothes.

Previous research considered the reasons that elderly Chinese people with hearing loss in Hong Kong do not seek out hearing aids [23]. Affordability was a key reason given by the 95 participants in that survey, with “cannot afford to buy a hearing aid” and “hearing aids are expensive” being the 5th and 9th most common reasons given. However, in the EasyHear participant group cost was rarely mentioned (by only four participants), suggesting that the price point for the device is seen as “affordable” by this group of elderly EasyHear clients. The study sample may be biased for this factor—less financially well-off individuals may not attend a private clinic such as EasyHear. On the other hand, the EasyHear Grand is a bilateral device and one instrument provides the same assistance as two conventional behind-the-ear or in-the-ear hearing aids, thus potentially reducing purchase costs.

### 4.5. Limitations

For this study only EasyHear Grand users without cognitive impairment were recruited. The effectiveness of the EasyHear Grand with cognitively challenged users is unknown. The study used standardized questionnaires that are widely used in rating client benefits and satisfaction with hearing aids. However, some of the questions were inappropriate for participants in this study (such as the C-PHAB reverberation items) and this limited the dataset obtained in the study. The open-ended interview used a broad but restricted question set—a less structured interview may have gained more insights into client attitudes and feelings about the EasyHear Grand. Client outcomes for the EasyHear Grand fittings were assessed within an eight-week timeframe. A longer term study—for example, over a 12-month period—may better map client outcomes postfitting. The majority of participants were elderly persons who may require longer fitting periods to fully adapt to hearing aid use.

A main feature of the EasyHear Grand device is its beamforming microphone function. However, the present research study was not designed to assess beamforming capabilities. Beamforming technology has potential advantages over conventional microphone designs to reduce competing noise and enhance speech in everyday listening situations. Research comparing speech recognition scores in an elderly client group for different microphone, noise, and environment parameters could quantify the effectiveness of this type of device technology.

## 5. Conclusions

Overall, the study participants expressed clear satisfaction with their EasyHear Grand device. Average ratings with the EasyHear Grand were equivalent to those achieved in other professional hearing aid fittings. For example, participants rated their EasyHear device fitting highly for hours of use, perceived benefit, satisfaction with the fitting outcome, and improved quality of life using the International Orientation Inventory-Hearing Aid questionnaire. Similarly, wearers rated the EasyHear positively for improved communication and benefit in background noise with the Profile of Hearing Aid Benefit questionnaire. The Client Oriented Scale of Improvement results showed that a large majority of EasyHear Grand users (81%) felt the device had greatly improved their listening ability in their main situation of need and also for their second listening priority (72%). Less than 10% of users felt their listening was only occasionally or hardly ever improved when using the EasyHear Grand. Participants gave a wide range of comments during their open-ended interview sessions. Nearly all the participants (41 out of 43) felt the device improved their listening ability. Nearly half the study participants had prior hearing aid experience and virtually all of this group considered that the EasyHear had improved sound quality compared to their previous hearing aid (20 out of 22 participants). The noise reduction performance of the EasyHear was valued by many participants. Interviews also highlighted areas where users felt the device could be improved—extra noise reduction, reduced device size, earphone/earmold design, wireless technology, and a pocket clip were among the items mentioned. Many of the senior clients in this study were “technology aware”—interview comments indicated that many of the elderly clients valued bilateral smartphone linkage and Bluetooth capability. Few participants expressed negative comments directly related to the body-worn form factor of the hearing aid (although wireless linkage was often suggested for future models), and none expressed a desire to wear an alternative BTE/ITE design.

The study findings suggest that the EasyHear Grand, with its body-worn format and simple controls, provides a level of benefit and satisfaction that is comparable to that reported for persons with hearing loss professionally fitted with a range of conventional design form hearing aids. The majority of elderly users in the present study expressed positive outcomes with the hearing aid fitting. Conventional hearing aid design formats may not be optimal for all elderly individuals with hearing impairment and innovative designs—such as those used in the EasyHear Grand—deserve consideration. An increased range of hearing aid design options may raise the currently low level of hearing aid usage in elderly populations.

## Figures and Tables

**Table 1:** Participant visit schedule and procedures.

	Visit 1	Visit 2	Visit 3

Time*∗*	Week 1	Week 2	Week 8

Facilitator	Audiologist	Research Assistant	Research Assistant

Patient information and consent form	✓		
Fit the device	✓		
Fine tune the device		✓	✓
Questionnaire (MoCA)	✓		
Questionnaire (IOI-HA)	✓	✓	✓
Questionnaire (C-PHAB)	✓	✓	✓
Questionnaire (COSI)	✓	✓	✓
Open-ended interview on continued usage			✓

Note:  *∗*: the time window for participant clinic visits was ± 1 week of target visit schedule.

**Table 2:** Gender, age, and hearing thresholds of study participants.

Group characteristics	
Number of participants	43
Male:Female ratio	28:15
First time hearing aid user (Yes:No:No data)	17:22:4
Mean age in years (SD)	71 (11)
Age range in years	46 - 88
Type of hearing loss right ear (Sensorineural:Mixed:No data)	28:14:1
Type of hearing loss left ear (Sensorineural:Mixed:No data)	31:11:1
Mean (range) hearing threshold right ear (dBHL)	69 (45 – 118)
Mean (SD) hearing threshold at 500 Hz right ear (dBHL)	64 (19)
Mean (SD) hearing threshold at 1000 Hz right ear (dBHL)	69 (17)
Mean (SD) hearing threshold at 2000 Hz right ear (dBHL)	73 (13)
Mean (SD) hearing threshold at 4000 Hz right ear (dBHL)	80 (17)
Mean (range) hearing threshold left ear (dBHL)	65 (42 - 118)
Mean (SD) hearing threshold at 500 Hz left ear (dBHL)	59 (14)
Mean (SD) hearing threshold at 1000 Hz left ear (dBHL)	67 (15)
Mean (SD) hearing threshold at 2000 Hz left ear (dBHL)	69 (14)
Mean (SD) hearing threshold at 4000 Hz left ear (dBHL)	76 (14)

*Note.* Threshold mean = 500 Hz, 1000 Hz, 2000 Hz, and 4000 Hz average.

**Table 3:** IOI-HA scores for all question items for three visits.

Question item	Visit	Score (All subjects)	Score (Prior HA use) n=22	Score (No prior HA use) n=17
Use	First	3.60	3.77	3.24

Use	Second	3.79	3.86	3.53

Use	Third	3.67	3.86	3.35

Benefit	First	3.30	3.59	2.94

Benefit	Second	3.84	3.86	3.88

Benefit	Third	3.93	4.05	3.88

Residual difficulty	First	3.67	3.68	3.76

Residual difficulty	Second	3.74	3.86	3.71

Residual difficulty	Third	3.70	3.82	3.65

Satisfaction	First	3.28	3.45	3.06

Satisfaction	Second	3.86	4.09	3.65

Satisfaction	Third	3.95	4.23	3.76

Residual participation restriction	First	3.79	3.86	3.76

Residual participation restriction	Second	3.86	4	3.82

Residual participation restriction	Third	3.88	4.05	3.88

Impact on others	First	4.02	4.18	3.88

Impact on others	Second	3.95	4.05	4

Impact on others	Third	3.93	4.05	3.82

Quality of life change	First	3.51	3.73	3.24

Quality of life change	Second	3.79	3.95	3.65

Quality of life change	Third	3.95	4.05	3.88

Mean overall score	First	3.59	3.74	3.41

Mean overall score	Second	3.83	3.95	3.75

Mean overall score	Third	3.86	4.01	3.75

ANOVA comparison of mean scores over three sessions		F(2,80)=10.53, p<.001*∗∗*	F(2,40)=4.34, p=.02*∗∗*	F(2,30)=9.22, p<.001*∗∗*

**Table 4:** C-PHAB scores for all question domains for three visits.

Domain		Valid N	Missing	Mean	Range
Ease of communication	Unaided	22	21	0.54	0.94
	Aided (Visit 1)	20	23	0.22	0.37
	Aided (Visit 2)	21	22	0.15	0.57
	Aided (Visit 3)	15	28	0.12	0.4
	Benefit (Visit 1)	20	23	0.33	0.86
	Benefit (Visit 2)	18	25	0.45	1.1
	Benefit (Visit 3)	14	29	0.47	0.88

Background noise	Unaided	34	9	0.58	0.83
	Aided (Visit 1)	27	16	0.27	0.47
	Aided (Visit 2)	30	13	0.25	0.72
	Aided (Visit 3)	32	11	0.22	0.56
	Benefit (Visit 1)	27	16	0.35	0.77
	Benefit (Visit 2)	26	17	0.34	0.95
	Benefit (Visit 3)	27	16	0.38	1.15

Reverberation	Unaided	9	34	0.77	0.53
	Aided (Visit 1)	9	34	0.27	0.5
	Aided (Visit 2)	5	38	0.16	0.37
	Aided (Visit 3)	2	41	0.13	0.2
	Benefit (Visit 1)	9	34	0.5	0.86
	Benefit (Visit 2)	5	38	0.59	0.45
	Benefit (Visit 3)	2	41	0.46	0.07

Aversiveness	Unaided	28	15	0.22	0.78
	Aided (Visit 1)	25	18	0.29	0.68
	Aided (Visit 2)	34	9	0.23	0.76
	Aided (Visit 3)	36	7	0.21	0.74
	Benefit (Visit 1)	25	18	-0.07	1.1
	Benefit (Visit 2)	25	18	0.04	1.23
	Benefit (Visit 3)	26	17	0.04	1.06

Global score	Unaided	9	34	0.72	0.62
	Aided (Visit 1)	9	34	0.25	0.29
	Aided (Visit 2)	5	38	0.15	0.21
	Aided (Visit 3)	2	41	0.1	0.14

**Table 5:** COSI specific needs nominated by participants at Visit 1.

	Face-to-face conversation	Telephone conversation	Speech in noise/Noise reduction	Distance listening	Watching television	Audibility to environmental signals	Do not have this need
Specific need 1	29 (67.4%)	4 (9.3%)	1 (2.3%)	5 (11.6%)	3 (7.0%)	1 (2.3%)	0
Specific need 2	5 (11.6%)	6 (14.0%)	13 (30.2%)	2 (4.7%)	8 (18.6%)	3 (7.0%)	1 (2.3%)
Specific need 3	2 (4.7%)	5 (11.6%)	4 (9.3%)	1 (2.3%)	6 (14.0%)	3 (7.0%)	18 (41.9%)
Specific need 4	0	1 (2.4%)	0	0	0	0	40 (95.2%)
Total	36 (83.7%)	16 (37.3%)	18 (41.8%)	8 (18.6%)	17 (39.6%)	7 (16.3%)	

*Note.* Needs nominated by less than 15% of participants are not shown.

**Table 6:** COSI specific needs outcome changes at Visits 2 and 3, categorized by main expressed needs and prioritized needs.

				Final ability				
		Participant number	Missing/Not applicable	Hardly ever: Improved 10% of time	Occasionally: Improved 25% of time	Half the time: Improved 50% of time	Most of the time: Improved 75% of time	Almost always: Improved 95% of time

Face-to-face conversation	Visit 2	36	0	0 (0%)	3 (8.3%)	7 (19.4%)	15 (41.7%)	11 (30.6%)
	Visit 3	36	0	1 (2.8%)	1 (2.8%)	4 (11.1%)	16 (44.4%)	14 (38.9%)
Speech in noise	Visit 2	18	1	1 (5.6%)	5 (27.8%)	5 (27.8%)	5 (27.8%)	1 (5.6%)
	Visit 3	18	3	1 (5.6%)	5 (27.8%)	5 (27.8%)	5 (27.7%)	2 (11.1%)
Phone conversation	Visit 2	15	2	0 (0%)	0 (0%)	0 (0%)	5 (33.3%)	8 (53.3%)
	Visit 3	15	1	0 (0%)	0 (0%)	0 (0%)	5 (33.3%)	9 (60%)

Specific need 1	Visit 2	42	1	0 (0%)	4 (9.5%)	6 (14.3%)	16 (38.1%)	16 (38.1%)
	Visit 3	42	1	2 (4.8%)	1 (2.4%)	5 (11.9%)	18 (42.9%)	16 (38.1%)
Specific need 2	Visit 2	39	4	2 (5.1%)	4 (10.3%)	7 (17.9%)	16 (41%)	10 (25.6%)
	Visit 3	39	4	2 (5.1%)	3 (7.7%)	6 (15.4%)	16 (41%)	12 (30.7%)
Specific need 3	Visit 2	24	19	2 (8.3%)	3 (12.5%)	6 (25%)	8 (33.3%)	5 (20.8%)
	Visit 3	23	20	2 (8.7%)	4 (17.4%)	3 (13%)	8 (34.8%)	6 (26.1%)

**Table 7:** Participant interview responses at Visit 3.

	*EH sounds clearer*	*EH noise reduction better*	*EH sounds more natural*	*EH has less echo & feedback*	*EH VC is user friendly*	*EH gives better sound localization*	*EH audibility is worse*	*EH amplifies own voice, noisier*	
Q1: Compare EH with previously used HA	20	13	9	4	4	2	1	1	

	*EH improves audibility*	*EH improves sound localization*	*Not much difference*						
Q2: Compare life with EH and without amplification	41	7	2						

	*Better audibility*	*Speech clearer*	*Phone function*	*Bluetooth function*	*Noise reduction*	*VC can be adjusted*	*Watching TV*	*Better sound localization*	*Radio function*
Q3: Specific benefit you like about EH	28	27	14	11	11	7	7	7	5

	*Remove the wire*	*Noise reduction not good enough *	*Receiver easy to fall *	*Device too big*	*Bluetooth function*	*TV and music unclear*	*Earmould discomfort*	*Not enough audibility*	
Q4: EH improvements suggested	28	10	7	7	7	5	5	4	

	*Good for noise reduction*	*Unaware of function*	*Not good enough for noise reduction*	*No difference*					
Q5: What do you think about beamforming?	14	9	9	7					

*Note.* Numbers refer to the number of participants who made this comment; EH = EasyHear Grand; HA = hearing aid; VC = volume control.

**Table 8:** IOI-HA scores for all question items for EasyHear Grand (Visit 3) and previous research.

Question item	Score
*Use* EasyHear Grand	3.67
*Use* HK 2005^*∗*^	3.26
*Use* USA 2003^†^ mild-moderate loss	3.73
*Use* USA 2003^†^ mod-severe loss	4.50

*Benefit *EasyHear Grand	3.93
*Benefit* HK 2005^*∗*^	3.53
*Benefit* USA 2003^†^ mild-moderate loss	3.39
*Benefit* USA 2003^†^ mod-severe loss	3.52

*Residual difficulty* EasyHear Grand	3.70
*Residual difficulty* HK 2005^*∗*^	4.42
*Residual difficulty* USA 2003^†^ mild moderate loss	3.40
*Residual difficulty* USA 2003^†^ mod-severe loss	3.19

*Satisfaction* EasyHear Grand	3.95
*Satisfaction* HK 2005^*∗*^	3.32
*Satisfaction* USA 2003^†^ mild-moderate loss	3.20
*Satisfaction* USA 2003^†^ mod-severe loss	3.84

*Residual participation restriction* EasyHear Grand	3.88
*Residual participation restriction* HK 2005^*∗*^	4.21
*Residual participation restriction* USA 2003^†^ mild-moderate loss	3.57
*Residual participation restriction* USA 2003^†^ mod-severe loss	3.38

*Impact on others* EasyHear Grand	3.93
*Impact on others* HK 2005^*∗*^	4.68
*Impact on others* USA 2003^†^ mild-moderate loss	3.79
*Impact on others* USA 2003^†^ mod-severe loss	3.38

*Quality of life change* EasyHear Grand	3.95
*Quality of life change* HK 2005^*∗*^	3.32
*Quality of life change* USA 2003^†^ mild-moderate loss	3.19
*Quality of life change* USA 2003^†^ mod-severe loss	3.68

*Mean overall score* EasyHear Grand	3.86
*Mean overall score* HK 2005^*∗*^	3.82
*Mean overall score* USA 2003^†^ mild-moderate loss	NA
*Mean overall score* USA 2003^†^ mod-severe loss	NA
*Mean overall score* Australia 2010^‡^ mod-severe loss	3.91

*Note.*  ^*∗*^McPherson & Wong (2005). Effectiveness of an affordable hearing aid with elderly persons. *Disability & Rehabilitation, 27*, 601-609. ^†^ Cox et al. (2003). Norms for the International Outcome Inventory for Hearing Aids. *Journal of the American Academy of Audiology, 10,* 67-79. ‡ Hickson, Clutterbuck & Khan (2010). Factors associated with hearing aid fitting outcomes on the IOI-HA. *International Journal of Audiology, 49,* 586-595. NA = not available.

## Data Availability

The data used to support the findings of this study are available from the corresponding author upon request.
